# Employment status and mortality among Korean men over a 13-year period

**DOI:** 10.4178/epih.e2021055

**Published:** 2021-08-18

**Authors:** Dohee Lim, Kyoung Ae Kong, Hyesook Park, Kyunghee Jung-Choi

**Affiliations:** 1 Department of Preventive Medicine, Ewha Womans University College of Medicine, Seoul, Korea; 2 Graduate Program in System Health Science and Engineering, Ewha Womans University, Seoul, Korea; 3 Department of Occupational and Environmental Medicine, Ewha Womans University College of Medicine, Seoul, Korea

**Keywords:** Employment status, Mortality, Precarious employee, Petty bourgeoisie

## Abstract

**OBJECTIVES:**

This study explored the effect of employment status on mortality over a 13-year period in Korean men.

**METHODS:**

Data were used from the Korean Labor and Income Panel Study from 1999 to 2012. This study started with 2,737 subjects and included employed men in good health, aged 30-69 years. Deaths were tracked for 13 years from 2000 to 2012. Employment status classifications were: (1) regular employees, (2) precarious employees, (3) petty bourgeoisie, and (4) employers. Hazard ratios (HRs) were calculated using a Cox proportional hazards model, and were adjusted for age, education, income, and occupation, with regular employees as the reference category. To examine the effect of employment status and include employment history, the risk ratios of mortality were measured using the Poisson regression model, considering the duration of each employment and using 0 years as the reference category.

**RESULTS:**

Over the course of the 13-year study, being a precarious employee (HR, 1.84) or petty bourgeoisie (HR, 1.87) at a particular point in time had a negative effect on mortality when compared with regular employees. Furthermore, working as precarious employees or petty bourgeoisie had no positive effect on mortality. A positive effect was observed, however, on the overall mortality risk for regular employees.

**CONCLUSIONS:**

These results suggest that a healthy social policy is needed for precarious employees and petty bourgeoisie to avoid disadvantages in the workplace and the social safety net.

## INTRODUCTION

Employment status is a socioeconomic position indicator that becomes even more important in an unstable labor market [1-3]. Although fragile employment status, such as precarious employment, has occurred widely in industrialized societies since the 19th century, the differentiation between the core and peripheral labor markets has been consolidated under neoliberalism. The number of precarious employees has increased, and their work and social environments have worsened, which is referred to as “polarization” [3-6].

Many studies have investigated the health status of precarious workers, with a primary focus on mental health [7-9]. Only a few European studies have explored the risk for mortality in precarious workers [10-13]. In Finland, temporary employees were found to have a 1.2-1.6 times increased mortality risk compared with permanent employees over 12 years [10] and men temporary workers in France had a 2.2 times increased mortality risk over 13 years [13]. In addition to studies that followed mortality by employment status at a single point in time, another study investigated the effect of employment history on mortality and confirmed a protective effect of employment itself on mortality [14].

Very few studies have explored the health risks of self-employment [15-17]. Self-employment can be defined as an employment status in which “remuneration is directly dependent upon the profits (or the potential for profits) derived from the goods and services produced” [18]. However, the self-employed are not a homogeneous group. Wright divided the self-employed into 3 groups according to the number of employees (capitalists, small employers, and petty bourgeoisie) [19], and the International Labor Office divided the self-employed into 2 groups (employers with employees and self-employed workers without employees) [18]. In order to survive in a competitive economic environment [15], petty bourgeoisie (or self-employed workers) are exposed to exploitation, a point that certainly warrants further study.

Korea underwent rapid industrialization after the Korean War in 1950-1953 [20], and adopted neoliberalism in the 1990s in order to change the economic and social environment [4, 6]. This change has necessitated exploration of the impact of social change on the vulnerable states of employment. This 13-year study explored the effect of employment status at a particular point in time, as well as the effect of employment history on mortality, in Korean men. We only included subjects who were healthy at the beginning of this study to avoid selection bias.

## MATERIALS AND METHODS

### Data

The data used in this study came from the Korean Labor and Income Panel Study (KLIPS), longitudinal survey of the labor market and income activities of a representative sample of Korean households and individuals living in urban areas. Wave 1 of the study began in 1998 with 5,000 households and their members (about 13,000 men and women aged ≥ 15 years). Wave 15 was completed in 2012, maintaining follow-up rates as high as 70-88% [21]. The KLIPS has been conducted annually to track the characteristics of households as well as their economic activities, labor movement, income, expenditures, education, job training, and social activities. The KLIPS has also investigated the death rate of study participants annually since the wave 2 study.

This study was able to generate a survival dataset using data from the KLIPS wave 2 and wave 15 studies for employed men aged 30-69 years, because a questionnaire on subjective health status was included in wave 2. Only those in good health were included at the beginning of the study to correct for selection bias. Of the 2,932 subjects, 195 (6.7%) workers whose subjective health status was poor were excluded. The percentage of excluded subjects was high in the petty bourgeoisie (10.6%), precarious employees (9.7%), 60-69 year-olds (20.1%), elementary school graduate or less (17.0%), skilled agricultural workers, forestry and fishery workers (20.6%), and those with low income (14.6%). After excluding those with poor subjective health status or missing employment values, 2,737 men were included in 1999 and deaths were tracked from 2000 to 2012. This study did not include women because of the low number of deaths and limited data.

### Variables

Employment status classifications were divided into regular employees, precarious employees, petty bourgeoisie, and employers. All temporary or daily workers, contingent workers, parttime workers, workers in temporary help agencies, workers provided by contract firms, home-based workers, on-call workers, and independent contractors were classified as precarious employees [22]. Regular and full-time workers were classified as regular employees. Among the self-employed, respondents who employed 1 or more workers were classified as employers and those who did not hire any workers were classified as petty bourgeoisie.

The levels of education were categorized by the International Standard Classification of Education (ISCED) as elementary school graduate or less (ISCED 1), middle school graduate (ISCED 2), high school graduate (ISCED 3), and college graduate or higher (ISCED 5); ISCED 4 was not included in this study because no equivalent level exists in Korea [23]. Occupational groups were classified into 9 categories according to the Korean Standard Classification of Occupations (KSCO): KSCO 0-1=legislators, senior officials, and managers, and professionals; KSCO 2=technicians and associate professionals; KSCO 3=clerks; KSCO 4=service workers; KSCO 5=sale workers; KSCO 6=skilled agricultural, forestry, and fishery workers; KSCO 7=craft and related trades workers; KSCO 8=plant and machine operators and assemblers; and KSCO 9=elementary occupations. Income was measured by equivalized household monthly income, using a square root scale that divides household income by the square root of household size. Participants were divided into income quartiles. Subjective health status was self-rated, based on a subject’s response to the question “How is your health in general? Would you say it is…?” Those who reported “very good”, “good”, or “moderate” were included in this study as having a good subjective health status and those who responded “bad” or “very bad” were excluded as having a poor subjective health status.

### Statistical analysis

Age-standardized mortality rates were calculated per 1,000 person-years based on direct standardization methods and using the World Health Organization world population as the standard population. The age-standardized mortality rates and 95% confidence intervals (CIs) were calculated by applying individual weights using the PROC STDRATE in SAS software.

To calculate the effect of employment status on mortality at the beginning of the study, the hazard ratios (HRs) were calculated using the Cox proportional hazards model, and were adjusted for age, educational level, equivalized household monthly income, and occupational group, with regular employees as the reference category. Model 1 was adjusted for age. In model 2, all variables included in the previous models were fully adjusted. In the sensitivity analysis, model 2 was further corrected for subjective health status to prevent selection bias.

To examine the effect of employment status on mortality, and also consider employment history, changes in employment status were tracked. Over the course of the study, the periods worked in each employment status were totaled, including unemployment and economic inactivity. The working period in each employment status was divided into 3 categories: 0 years, 1-4 years, and 5-13 years. Periods of unemployment were divided into 0 years and 1-13 years because those with more than 5 years of unemployment had low person-years and no deaths. The mortality risk ratios were measured using the Poisson regression model to include the duration of each employment status, unemployment, and economic inactivity, with 0 years as the reference category. In the Poisson regression model, model 1 was adjusted for age, and model 2 was additionally adjusted for education, occupational group, and income.

The individual cross-sectional sampling weights for 1999 were used for the analysis to produce representative results. All estimates, such as age-standardized mortality rates, HRs, and risks ratios were calculated by applying these weights. These analyses were conducted using SAS version 9.3 (SAS Institute Inc., Cary, NC, USA) and the PROC STDRATE, PROC PHREG, and PROC GENMOD procedures. The survival function was estimated using the age-adjusted Cox proportional hazards model, and the coxph() and survfit() functions were used in R version 3.3.2 (https://cran.r-project.org/).

### Ethics statement

This study was approved by the Ewha Medical Center Institutional Review Board, Seoul, Korea (IRB FILE No. 2015-04-038).

## RESULTS

Table 1 shows the characteristics of the study subjects by employment status in 1999. Of the 2,737 subjects, 48.1% were regular employees, followed by petty bourgeoisie (23.8%), precarious employees (14.6%), and employers (13.5%). Precarious employees and petty bourgeoisie tended to be older, had lower education levels, and had lower household income than regular employees and employers. Among regular employees, plant and machine operators and assemblers (22.9%) and clerks (22.2%) were the main occupations, while among precarious employees those occupations accounted for only 10.5% and 5.0%, respectively. The main occupations among precarious employees were craft and related trades (40.1%) and elementary occupations (27.6%). Almost half of the petty bourgeoisie were service workers or skilled agricultural, forestry, or fishery workers.

**Figure 1.**
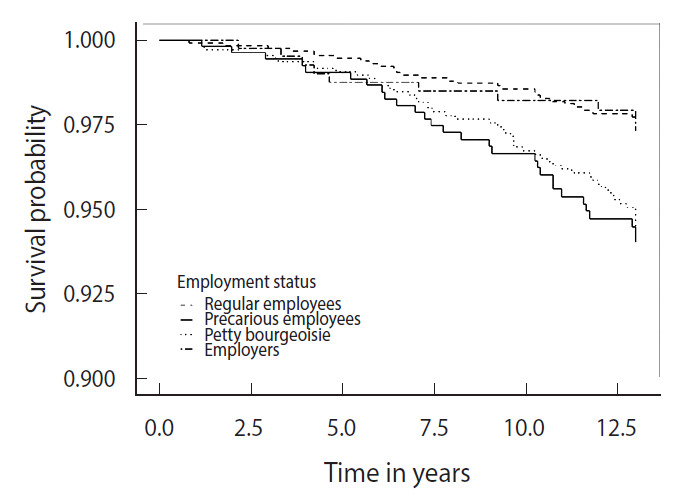
Survival graphs^1^ for mortality for 13 years by employment status in 1999. ^1^Adjusted for age.

Table 2 shows the age-standardized mortality rates according to employment status and other socioeconomic position indicators. By the end of the mortality follow-up period, 122 (4.5%) workers had died, and the observed study participants represented 30,526.1 person-years in total. The total mortality rate during 2000-2012 was 5.54 (95% CI, 5.52 to 5.56) per 1,000 person-years. The petty bourgeoisie had the highest mortality rate of 7.63 (95% CI, 7.59 to 7.68) per 1,000 person-years; followed by precarious employees with 6.43 (95% CI, 6.38 to 6.48), regular employees with 3.95 (95% CI, 3.91 to 3.99), and employers with 2.57 (95% CI, 2.54 to 2.61) per 1,000 person-years. The mortality rate of skilled agricultural, forestry, and fishery workers was the highest at 8.77 (95% CI, 8.69 to 8.84) per 1,000 person-years, whereas plant and machine operators and assemblers had the lowest mortality rate at 2.21 (95% CI, 2.18 to 2.24) per 1,000 person-years. Lower levels of education and income were associated with higher mortality rates. Men with elementary school or lower education and those in the lowest income quartile had the highest mortality rates (over 8 per 1,000 person-years). Survival graphs for the observed age-adjusted survival times by employment status are presented in Figure 1. The survival probabilities of precarious employees and petty bourgeoisie were lower than those of regular employees and employers.

Table 3 shows the HRs for mortality in each group defined by employment status. In model 1, the mortality risks for precarious employees and petty bourgeoisie, when considering age alone, were 2.53 (95% CI, 1.50 to 4.26) and 2.25 (95% CI, 1.41 to 3.60) times higher than that of regular employees, respectively. The risk for employers was 1.10 (95% CI, 0.54 to 2.26) times higher than that of regular employees, but the difference was not statistically significant. In model 2, the mortality risks for precarious employees and petty bourgeoisie were 1.84 (95% CI, 1.03 to 3.28) and 1.87 (95% CI, 1.04 to 3.38) times higher than that of regular employees, respectively, after adjusting for education level, occupational group, and income simultaneously.

Table 4 presents the mortality risk ratios according to the duration of each employment status. After adjusting for age and other employment status factors, regular employees working for 1-4 years had a lower mortality risk than those without a regular work history. Regular employees working for 5-13 years tended to have lower mortality risk ratios than those working for 1-4 years, although this result was not statistically significant. This was also true in the model adjusted for age, other employment status, education level, occupational group, and income. The trend was similar for employers. No decreasing trend in mortality risk was observed for precarious employees or petty bourgeoisie. A population that was economically inactive for 1 or more years had a higher mortality risk than those without an inactive economic history, whereas those with an unemployment history showed a lower mortality risk than those without an unemployment history.

### Sensitivity analysis

The subjective health status at the start of the study was adjusted, and the same analysis used in Table 3 was performed to further distinguish the effect of this selection criterion from the effect of employment status on death. When initial subjective health status was further considered, the risk of death for precarious employees and employers increased slightly, while the risk for petty bourgeoisie decreased slightly. However, the changes were small and statistical significance did not change (Supplementary Material 1).

## DISCUSSION

This study revealed that belonging to the categories of precarious employees or petty bourgeoisie had a negative effect on mortality and that being a regular employee had a positive effect on mortality when analyzed at a particular point in time over 13 years. The mortality risk for precarious employees and petty bourgeoisie was significantly higher than for regular employees, indicating that the characteristics of their employment status throughout the 13-year study were unfavorable in comparison with regular employees.

Higher mortality of precarious or temporary workers compared with regular employees has been reported in Finnish and French studies [10,12,13] and those results are consistent with this study. The mortality risk for temporary workers in France was found to be 2.21 times higher than for regular employees [13], based on employment status at the start of the study. The self-rated health of precarious employees was worse than that of regular employees in a previous study in Korea, which is also in line with the results of this study [24].

The higher mortality risk for precarious employees when compared with regular employees is likely attributable to 2 different mechanisms: the hazards of working as a precarious employee and the added insults of subsequent unfavorable employment [25]. First, the nature of precarious work lends itself to a higher risk of exposure to health hazards that could then lead to higher mortality risk, including hazardous physical and psychosocial working conditions with job insecurity [1,24], as well as social and material deprivation with or without a social safety net [4,26]. Second, working as a precarious employee could affect a person’s next step in the labor market [27]. For example, the economic circumstances of regular employees were closely related to whether they might enter into the petty bourgeoisie. Poorer economic conditions increased the likelihood of a transition to the petty bourgeoisie [28]. However, precarious employees inherently have unstable employment, and they frequently entered the petty bourgeoisie category regardless of their economic circumstances.

The proportion of petty bourgeoisie in Korea is on a declining trend over the long term, but is still high compared to other Organization for Economic and Cooperation Development countries. According to the 2013 Economically Active Population Survey, 16.7% of employed individuals were classified as petty bourgeoisie in Korea [29]. In our study, the mortality risk of petty bourgeoisie was higher than that of regular employees, but similar to that of precarious employees. No other studies were found that compared mortality among petty bourgeoisie to that of regular employees. Several studies have reported on the mortality risk of the self-employed. However, they did not differentiate petty bourgeoisie from employers [30,31], or compare the mortality of petty bourgeoisie with paid employees [32] or capitalists [33,34].

As with precarious employees, the finding that petty bourgeoisie experienced higher mortality than regular employees can be attributed to two different mechanisms. First, the risk of exposure to health hazards for petty bourgeoisie is likely higher than for regular employees. Although the self-employed have higher autonomy and subjectivity at work when compared with other employees, they work longer hours, have a heavier workload, and are more exposed to uncertainty, market fluctuations, and the threat of asset loss [15]. High autonomy can be a factor that positively affects health, but under competitive and uncertain economic pressure, high autonomy might result in longer working hours that exploit their own health as well as that of their labor force. The petty bourgeoisie in Korea were also more likely to be poorly educated and older, with low income, high employment instability, and the longest working hours, placing them at higher risk for objective and subjective health problems [28]. In addition, they have been excluded from the social security system, which offers access to industrial accident compensation insurance. This may have negatively contributed to the long-term health of the petty bourgeoisie, leading to a higher mortality risk than that of regular employees. In Korea, the self-employed are not required to obtain industrial accident compensation insurance. Employers with ≤ 50 employees can obtain it voluntarily, but if the self-employed do not have employees, they are not eligible for industrial accident compensation insurance [35]. The National Health Insurance program in Korea is a compulsory social insurance program that covers the entire population. Therefore, the greater difficulty for a sick or injured worker is managing the cost of living during treatment, rather than the cost of the medical treatment itself. Industrial accident compensation insurance helps to solve these difficulties by providing support for treatment fees, the cost of living during treatment, disability benefits in the case of disability, and survivor benefits in the case of death. However, as the petty bourgeoisie are excluded from industrial accident compensation insurance, they must bear these burdens themselves.

Second, those who began as petty bourgeoisie at the beginning of this study and later transitioned to precarious employee status had worse overall health outcomes than regular employees. In Korea, only 0.3% of the petty bourgeoisie are covered by employment insurance [35]. This means that when petty bourgeoisie go out of business, they cannot receive unemployment benefits. This often leads to a worse working environment following the closure of a business.

In this study, cumulative work as a regular employee was observed to have a positive effect on mortality. This trend was similar in employers. However, a positive effect from cumulative work as a precarious employee or petty bourgeoisie was not observed. Working in the labor market can be good or bad for an individual’s health. The health effect of working is presented as the net effect of both positive [36] and negative effects of employment. Our study suggests that the positive effect of employment was not greater than the negative effect among precarious employees and petty bourgeoisie.

This study had some limitations. First, this study did not consider cause of death when analyzing the differences in mortality rates. Second, because of limitations in the data, women were excluded from the study. Third, the effect of the workplace size on mortality rates could not be considered due to limitations in the data. Employers are expected to have heterogeneous characteristics based on the size of the workplace, which could lead to an overestimate of the mortality risk of employers compared to that of regular employees. Fourth, the original sample retention rate of the KLIPS data up to wave 15 used in our study was 70.3%. Studies [37,38] of KLIPS attrition have shown that high household income and low individual income are associated with a higher probability of sample drop-out, meaning that the measure of inequality calculated by KLIPS can underestimate the degree of inequality [38]. Due to these limitations inherent to the KLIPS data, mortality according to employment status as measured in our study may have been underestimated. Fifth, when analyzing the employment histories, only the duration of employment status was considered and the order of change in employment status was not taken into account. This is an area that needs further analysis.

This study also had some important strengths. First, we excluded subjects with poor health status to reduce selection bias. Selection bias should be a concern because health could be a factor in determining employment status [17]. Second, this study explored the mortality risk of petty bourgeoisie and precarious employees using follow-up data.

In conclusion, this study demonstrated that belonging to the categories of precarious employees or petty bourgeoisie had negative effects on mortality when compared with regular employees at a particular point in time over a 13-year period. No positive effect of working was found for precarious employees or petty bourgeoisie, whereas cumulative work as a regular employee was observed to have a positive effect on mortality. This study suggests that a healthy social policy for precarious employees and petty bourgeoisie is needed to avoid disadvantages in the workplace and the social safety net.

## Figures and Tables

**Table 1.** Characteristics of study subjects by employment status among employed men aged 30-69 years in 1999

Characteristics	Regular employees	Precarious employees	Petty bourgeoisie	Employers	Total
Total	1,317 (48.1)	399 (14.6)	652 (23.8)	369 (13.5)	2,737 (100)
Age (yr)					
30-39	649 (49.3)	145 (36.3)	164 (25.2)	137 (37.1)	1,095 (40.0)
40-49	422 (32.0)	125 (31.3)	229 (35.1)	152 (41.2)	928 (33.9)
50-59	202 (15.3)	96 (24.1)	171 (26.2)	62 (16.8)	531 (19.4)
60-69	44 (3.3)	33 (8.3)	88 (13.5)	18 (4.9)	183 (6.7)
Education					
Elementary school graduate or less	89 (6.8)	94 (23.6)	160 (24.5)	13 (3.5)	356 (13.0)
Middle school graduate	167 (12.7)	88 (22.1)	144 (22.1)	42 (11.4)	441 (16.1)
High school graduate	549 (41.7)	165 (41.4)	277 (42.5)	175 (47.4)	1,166 (42.6)
College graduate or higher	512 (38.9)	52 (13.0)	71 (10.9)	139 (37.7)	774 (28.3)
Occupation					
Legislators, senior officials and managers, professionals	180 (13.7)	16 (4.0)	11 (1.7)	26 (7.0)	233 (8.5)
Technicians and associate professionals	184 (14.0)	20 (5.0)	27 (4.1)	42 (11.4)	273 (10.0)
Clerks	292 (22.2)	20 (5.0)	24 (3.7)	66 (17.9)	402 (14.7)
Service workers	42 (3.2)	14 (3.5)	57 (8.7)	38 (10.3)	151 (5.5)
Sale workers	44 (3.3)	13 (3.3)	155 (23.8)	68 (18.4)	280 (10.2)
Skilled agricultural, forestry and fishery workers	3 (0.2)	4 (1.0)	159 (24.4)	11 (3.0)	177 (6.5)
Craft and related trades workers	163 (12.4)	160 (40.1)	82 (12.6)	77 (20.9)	482 (17.6)
Plant and machine operators and assemblers	301 (22.9)	42 (10.5)	108 (16.6)	31 (8.4)	482 (17.6)
Elementary occupations	108 (8.2)	110 (27.6)	29 (4.4)	10 (2.7)	257 (9.4)
Income					
Low	203 (15.4)	167 (41.9)	246 (37.7)	68 (18.4)	684 (25.0)
Lower middle	328 (24.9)	101 (25.3)	169 (25.9)	77 (20.9)	675 (24.7)
Upper middle	389 (29.5)	71 (17.8)	126 (19.3)	90 (24.4)	676 (24.7)
High	397 (30.1)	60 (15.0)	111 (17.0)	134 (36.3)	702 (25.6)

Values are presented as number (%).

**Table 2.** Age-standardized mortality rates (per 1,000 person-years) in 2000-2012 by employment status and other socioeconomic indicators among employed men aged 30-69 years in 1999

Variables	Person-year	No. of deaths	Age-standardized mortality	95% CI	
				UL	LL
Total	30,526.1	122	5.54	5.52	5.56
Employment status					
Regular employees	14,668.0	29	3.95	3.91	3.99
Precarious employees	4,436.8	29	6.43	6.38	6.48
Petty bourgeoisie	7,471.6	54	7.63	7.59	7.68
Employers	3,949.7	10	2.57	2.54	2.61
Education					
Elementary school graduate or less	4,163.7	43	8.46	8.40	8.52
Middle school graduate	5,034.8	30	5.65	5.61	5.70
High school graduate	13,066.1	35	3.99	3.96	4.03
College graduate or higher	8,261.5	14	2.88	2.84	2.91
Occupation					
Legislators, senior officials and managers, professionals	2,465.3	5	2.80	2.74	2.85
Technicians and associate professionals	2,849.2	7	4.28	4.22	4.34
Clerks	4,488.3	12	2.93	2.89	2.96
Service workers	1,678.7	7	7.52	7.36	7.68
Sales workers	3,072.3	10	4.98	4.90	5.05
Skilled agricultural, forestry and fishery workers	2,090.3	26	8.77	8.69	8.84
Craft and related trades workers	5,493.2	25	4.12	4.08	4.15
Plant and machine operators and assemblers	5,464.1	11	2.21	2.18	2.24
Elementary occupations	2,924.9	19	6.92	6.85	6.99
Income					
Low	7,500.3	51	8.48	8.43	8.53
Lower middle	7,549.8	27	5.71	5.66	5.76
Upper middle	7,650.8	24	4.20	4.16	4.24
High	7,825.1	20	3.25	3.22	3.28

CI, confidence interval; UL, upper limit; LL, lower limit.

**Table 3.** Adjusted hazard ratios of mortality in 2000-2012 according to employment status in men aged 30-69 years in 1999

Variables	Model 1^1^	Model 2^2^
Employment status		
Regular employees	1.00 (reference)	1.00 (reference)
Precarious employees	2.53 (1.50, 4.26)	1.84 (1.03, 3.28)
Petty bourgeoisie	2.25 (1.41, 3.60)	1.87 (1.04, 3.38)
Employers	1.10 (0.54, 2.26)	1.14 (0.55, 2.38)
Educational attainment		
Elementary school graduate or less	-	2.12 (0.99, 4.55)
Middle school graduate	-	2.08 (0.99, 4.33)
High school graduate	-	1.27 (0.64, 2.56)
College graduate or higher	-	1.00 (reference
Occupational group		
Legislators, senior officials and	-	1.00 (reference)
managers and professionals		
Technicians and associate professionals	-	0.94 (0.29, 3.03)
Clerks	-	1.13 (0.38, 3.36)
Service workers	-	0.83 (0.24, 2.90)
Sale workers	-	0.62 (0.19, 2.02)
Skilled agricultural, forestry and	-	0.87 (0.28, 2.69)
fishery workers		
Craft and related trades workers	-	0.97 (0.33, 2.88)
Plant and machine operators and	-	0.54 (0.17, 1.69)
assemble's		
Elementary occupations	-	0.86 (0.28, 2.59)
Income		
Low	-	1.83 (1.05, 3.17)
Lower middle	-	1.20 (0.66, 2.19)
Upper middle	-	1.24 (0.68, 2.27)
High	-	1.00 (reference)

Values are presented as hazard ratio (95% confidence interval).

1 Adjusted for age.

2 Adjusted for age, educational attainment, occupational group and income.

**Table 4.** Mortality risk ratios in 2000-2012 according to the duration of each employment status in 1999-2011 in men aged 30-69 years in 1999

Work period (yr)	Person-years	No. of deaths	Model 1^1^	Model 2^1^
Regular employees				
0	13,861	81	1.00 (reference)	1.00 (reference)
1-4	10,738	24	0.41 (0.24, 0.69)	0.56 (0.33, 0.96)
5-13	8,049	17	0.35 (0.18, 0.66)	0.54 (0.28, 1.04)
Precarious employees				
0	23,413	78	1.00 (reference)	1.00 (reference)
1-4	7,192	30	0.81 (0.51, 1.28)	0.89 (0.55, 1.41)
5-13	2,043	14	0.91 (0.47, 1.78)	1.10 (0.55, 2.19)
Petty bourgeoisie				
0	20,507	60	1.00 (reference)	1.00 (reference)
1-4	7,776	31	0.85 (0.51, 1.41)	0.94 (0.55, 1.60)
5-13	4,365	31	1.19 (0.68, 2.10)	1.38 (0.76, 2.50)
Employers				
0	24,402	99	1.00 (reference)	1.00 (reference)
1-4	6,333	19	0.54 (0.32, 0.90)	0.63 (0.37, 1.07)
5-13	1,913	4	0.36 (0.13, 1.01)	0.56 (0.19, 1.58)
Unemployed				
0	30,185	115	1.00 (reference)	1.00 (reference)
1-13	2,463	7	0.43 (0.20, 0.95)	0.35 (0.16, 0.76)
Not economically active population				
0	27,177	53	1.00 (reference)	1.00 (reference)
1-4	4,457	56	6.03 (3.98, 9.15)	2.12 (1.05, 4.26)
5-13	1,014	13	3.81 (1.88, 7.76)	1.14 (0.45, 2.89)

Values are presented as hazard ratio (95% confidence interval).

1 Adjusted for age and other employment status.

2 Adjusted for age, other employment status, educational attainment, occupational stratification, and income.

## SUPPLEMENTARY MATERIALS

Supplementary Material 1. Further correction of subjective health status for adjusted hazard ratios (95% confidence interval) of mortality according to employment status in male aged 30-69 years old
