# Supplementary material for: Employment status and mortality among Korean men over a 13-year period
Source: Epidemiol Health. 2021 Aug 18;43:e2021055. doi: 10.4178/epih.e2021055 (PMC8510830; doi:10.4178/epih.e2021055)
Supplement: Supplementary Material 1. — Further correction of subjective health status for adjusted hazard ratios (95% confidence interval) of mortality according to employment status in male aged 30-69 years old [file epih-43-e2021055-suppl.docx]

Supplementary Material 1. Further correction of subjective health status for adjusted hazard ratios (95% confidence interval) of mortality according to employment status in male aged 30-69 years old

|  | Model 1^*^ | Model 2^†^ | Model 3^‡^ | Model 4^§^ | Model 5^‖^ |
| --- | --- | --- | --- | --- | --- |
| Employment status |  |  |  |  |  |
| Regular employees | 1.00 (reference) | 1.00 (reference) | 1.00 (reference) | 1.00 (reference) | 1.00 (reference) |
| Precarious employees | 2.57 (1.53, 4.33) | 2.12 (1.24, 3.62) | 2.22 (1.26, 3.92) | 2.19 (1.29, 3.74) | 1.88 (1.06, 3.35) |
| Petty bourgeoisie | 2.23 (1.40, 3.56) | 1.87 (1.15, 3.04) | 2.17 (1.22, 3.87) | 1.95 (1.21, 3.15) | 1.85 (1.03, 3.33) |
| Employers | 1.13 (0.55, 2.31) | 1.16 (0.56, 2.37) | 1.12 (0.54, 2.34) | 1.13 (0.55, 2.33) | 1.15 (0.55, 2.40) |
| Educational attainment |  |  |  |  |  |
| Elementary school graduate or less |  | 2.19 (1.12, 4.28) |  |  | 2.14 (1.00, 4.58) |
| Middle school graduate |  | 2.05 (1.06, 3.98) |  |  | 2.12 (1.01, 4.43) |
| High school graduate |  | 1.25 (0.66, 2.36) |  |  | 1.29 (0.64, 2.58) |
| College graduate or higher |  | 1.00 (reference) |  |  | 1.00 (reference) |
| Occupational group |  |  |  |  |  |
| Legislators, senior officials and managers and professionals |  |  | 1.00 (reference) |  | 1.00 (reference) |
| Technicians and associate professionals |  |  | 0.94 (0.29, 2.99) |  | 0.88 (0.27, 2.85) |
| Clerks |  |  | 1.28 (0.45, 3.64) |  | 1.04 (0.35, 3.12) |
| Service workers |  |  | 1.18 (0.36, 3.91) |  | 0.81 (0.23, 2.85) |
| Sale workers |  |  | 0.89 (0.29, 2.78) |  | 0.60 (0.18, 1.98) |
| Skilled agricultural, forestry and fishery workers |  |  | 1.41 (0.48, 4.12) |  | 0.83 (0.27, 2.60) |
| Craft and related trades workers |  |  | 1.52 (0.56, 4.10) |  | 0.95 (0.32, 2.82) |
| Plant and machine operators and assemblers |  |  | 0.76 (0.26, 2.22) |  | 0.51 (0.16, 1.61) |
| Elementary occupations |  |  | 1.40 (0.50, 3.88) |  | 0.81 (0.27, 2.45) |
| Income |  |  |  |  |  |
| Low |  |  |  | 1.99 (1.16, 3.39) | 1.74 (1.00, 3.03) |
| Lower middle |  |  |  | 1.31 (0.73, 2.36) | 1.21 (0.66, 2.19) |
| Upper middle |  |  |  | 1.28 (0.70, 2.31) | 1.24 (0.68, 2.26) |
| High |  |  |  | 1.00 (reference) | 1.00 (reference) |
| Subjective health status |  |  |  |  |  |
| Very good | 1.00 (reference) | 1.00 (reference) | 1.00 (reference) | 1.00 (reference) | 1.00 (reference) |
| Good | 0.77 (0.50, 1.19) | 0.74 (0.48, 1.15) | 0.77 (0.50, 1.20) | 0.78 (0.50, 1.21) | 0.76 (0.49, 1.18) |
| Normal | 1.29 (0.81, 2.06) | 1.26 (0.79, 2.01) | 1.30 (0.81, 2.08) | 1.24 (0.78, 1.98) | 1.22 (0.76, 1.96) |

^*^ Adjusted by age, subjective health status; ^†^ Adjusted by age, subjective health status, educational attainment; ^‡^ Adjusted by age, subjective health status, occupational stratification; ^§^ Adjusted by age, subjective health status, income; ^‖^ Adjusted by age, subjective health status, educational attainment, occupational stratification, income.
